# Presence of Stromal Cells Enhances Epithelial-to-Mesenchymal Transition (EMT) Induction in Lung Bronchial Epithelium after Protracted Exposure to Oxidative Stress of Gamma Radiation

**DOI:** 10.1155/2019/4120379

**Published:** 2019-09-08

**Authors:** Anna Acheva, Siamak Haghdoost, Alice Sollazzo, Virpi Launonen, Meerit Kämäräinen

**Affiliations:** ^1^STUK-Radiation and Nuclear Safety Authority, Helsinki, Finland; ^2^Department of Veterinary Biosciences, Section of Pathology, Faculty of Veterinary Medicine, University of Helsinki, Finland; ^3^Department of Molecular Bioscience, Centre for Radiation Protection Research, The Wenner-Gren Institute, Stockholm University, Stockholm, Sweden; ^4^University of Caen Normandy, Cimap-Laria, Advanced Resource Center for HADrontherapy in Europe (ARCHADE), Caen, France

## Abstract

The aim of the study was to investigate the role of a microenvironment in the induction of epithelial-to-mesenchymal transition (EMT) as a sign of early stages of carcinogenesis in human lung epithelial cell lines after protracted low-dose rate *γ*-radiation exposures. BEAS-2B and HBEC-3KT lung cell lines were irradiated with low-dose rate *γ*-rays (^137^Cs, 1.4 or 14 mGy/h) to 0.1 or 1 Gy with or without adding TGF-*β*. TGF-*β*-treated samples were applied as positive EMT controls and tested in parallel to find out if the radiation has a potentiating effect on the EMT induction. To evaluate the effect of the stromal component, the epithelial cells were irradiated in cocultures with stromal MRC-9 lung fibroblasts. On day 3 post treatment, the EMT markers: *α*-SMA, vimentin, fibronectin, and E-cadherin, were analyzed. The oxidative stress levels were evaluated by 8-oxo-dG analysis in both epithelial and fibroblast cells. The protracted exposure to low Linear Energy Transfer (LET) radiation at the total absorbed dose of 1 Gy was able to induce changes suggestive of EMT. The results show that the presence of the stromal component and its signaling (TGF-*β*) in the cocultures enhances the EMT. Radiation had a minor cumulative effect on the TGF-*β*-induced EMT with both doses. The oxidative stress levels were higher than the background in both epithelial and stromal cells post chronic irradiation (0.1 and 1 Gy); as for the BEAS-2B cell line, the increase was statistically significant. We suggest that the induction of EMT in bronchial epithelial cells by radiation requires more than single acute exposure and the presence of stromal component might enhance the effect through free radical production and accumulation.

## 1. Introduction

Ionizing radiation has been pointed out as the second leading cause for lung cancer after smoking [1]. However, there are no detailed mechanistic models for the early stages of radiation-induced lung carcinogenesis so far.

Over the last fifteen years, the important role of the tissue microenvironment (stroma) for the early stages of carcinogenesis and particularly the role of microenvironment in radiation-induced breast cancer have been highlighted [2–5]. The stroma of the tissue provides the “soil” for the transformed cells to grow and invade distant from their origin causing metastasis. It also produces specific chemokines and signaling molecules such as transforming growth factor beta (TGF-*β*), epidermal growth factor (EGF), and hepatocyte growth factor (HGF) during tumorigenesis [2, 3].

An important step during the modification of the microenvironment is the induction of morphological and functional changes of the normal stromal fibroblasts. These activated growth factor- and chemokine-secreting fibroblast cells are very often associated with the tumor microenvironment and are thus called cancer-associated fibroblasts (CAFs) [6, 7]. They are considered to be able to induce tumor growth and dissemination via secretion of different factors, stimulate angiogenesis, and interact with the tumors' growth factor receptors. CAFs have a set of common characteristics that allows for easy distinguishing from normal fibroblasts. The majority of these CAF markers have been identified as various tropic molecules and extracellular matrix remodeling enzymes: alpha-smooth muscle actin (*α*-SMA), matrix metalloproteinase-1 (MMP1), matrix metalloproteinase-3 (MMP3), and collagens [8, 9]. The expression of some myofibroblast markers, such as *α*-SMA, can be used as predictive markers for cancer recurrence and distant metastasis [7].

In the last decades, a process taking part in the epithelial cells has been described—epithelial-to-mesenchymal transition (EMT) [10–12]. It has a role not only in organogenesis and wound healing but also in disease pathogenesis like fibrosis and cancer. During EMT under microenvironmental stimulation, the epithelial cells change their shape and lose their polarity as well as expression of epithelial markers. At the same time, they acquire a spindle-shaped mesenchymal phenotype and gain expression of mesenchymal markers [13].

One of the important pathways shared by EMT and tumorigenesis is the activation of the TGF-*β* signaling pathway [11–14]. The TGF-*β* molecule affects the tumor microenvironment as it decreases the levels of active immune system cells, increases angiogenesis, and facilitates invasion by enhancing the cellular protease activity and the production of extracellular matrix components by the tumor microenvironment cells. It is interesting from the radiation point of view that the TGF-*β* pathway is induced by oxidative stress, which is one of the main cell-damaging conditions produced by low LET radiation [15] particularly at a low-dose rate [16]. The connection between oxidative stress, TGF-*β* signaling, and the role of the microenvironment in radiation-induced cancer has been studied in detail for breast models [4, 5, 17]. It was also proven that low dose and low-dose rate gamma radiation at mGy/h range induces oxidative stress by increasing the endogenous production of reactive oxygen species in primary human fibroblast cells (VH10), whole blood samples, and human lymphocytes [18]. Exposure to ionizing radiation (IR) is regarded as a sensitizing factor for cells to undergo TGF-*β*-induced EMT. Andarawewa et al. [19, 20] showed that a single exposure to IR sensitizes cells to TGF-*β*-mediated EMT. Neither radiation nor a chronic TGF-*β* secretion alone could induce EMT [19–22]. Radiation-induced secretion of TGF-*β*, not from the epithelial cells themselves, but from the surrounding stroma may increase the occurrence of EMT, which could be one of the early stages in the radiation-activated tumorigenic changes [23].

The latent TGF-*β* activation due to reactive oxygen species (ROS) is so efficient that it can be used as a sensor for the oxidative stress [17]. TGF-*β* is also upregulated in a NSCLC (non-small-cell lung cancer) patient's blood samples during radiotherapy [24]. The high TGF-*β* levels have been connected not only with severe late effects but also with insufficient response to radiotherapy. The TGF-*β* signaling pathway has been known for many years to be involved in the tissue remodeling and induction of late effects of radiotherapy in the lung, as it has been considered one of the main mediators of tissue fibrosis in the organ [12, 25].

In this pilot project, we tested the hypothesis that radiation modifies the lung stromal cells, thus creating an environment that facilitates EMT and promotes tumorigenesis. Our aim was to investigate the role of the microenvironment in the induction of EMT in human lung epithelial cells after protracted low-dose rate *γ*-ray exposures, which so far has not been explored. Such type of protracted radiation exposures with low-dose rates could occur in real life for example to workers at uranium mines or to individuals living in near proximity of contaminated areas [26–28]. To our knowledge, this is the first of its kind study of lung epithelial cells and the questions we address need further and more detailed investigation in the future to reveal the mechanisms of radiation-induced lung carcinogenesis.

## 2. Materials and Methods

### 2.1. Cell Culture

The human bronchial epithelial cell line BEAS-2B was purchased from the American Type Culture Collection (ATCC, Manassas, VA, USA) and maintained in serum-free Bronchial Epithelial Cell Growth Medium (BEGM) medium (Lonza, Walkersville, MD, USA), supplemented with Bronchial Epithelial Cell Growth Medium SingleQuots™ Supplements and Growth Factors (Lonza). The HBEC-3KT bronchial epithelial cell line was kindly provided by Professor Jerry Shay (UT Southwestern, TX, USA). The cells were immortalized in the laboratory of Prof. Shay and further cultured as described by Ramirez et al. [29]. In brief, the cells were cultured in KSF (Keratinocyte Serum-Free) media (Gibco, Carlsbad, CA, USA) supplemented with epidermal growth factor 1-53 (EGF 1-53) and Bovine Pituitary Extract (BPE) provided frozen from the manufacturer. The MRC-9 normal human lung fibroblasts were purchased from the ATTC. The cells were expanded and maintained in Eagle's Minimum Essential Medium (Sigma-Aldrich, St. Louise, MO, USA) supplemented with fetal bovine serum and L-glutamine as instructed by the supplier.

The cells were stimulated with 0.1-0.2 ng/ml recombinant human TGF-*β*1 (#240-B-002, R&D Systems, Minneapolis, USA).

### 2.2. Coculturing of the Cells

For the coculture experiments, 1 × 10^4^ BEAS-2B or HBEC-3KT cells were plated on 0.42 cm^2^ polycarbonate membrane inserts with a 0.4 *μ*m pore size (Nunc, Thermo Fisher Scientific, Waltham, MA, USA) and placed into 35 mm plastic petri dishes (Corning, Tewksbury, MA, USA). For the coculturing, 1 × 10^5^ MRC-9 normal human lung fibroblasts were plated on the bottom of the 35 mm plastic petri dishes, and the standing inserts, containing BEAS-2B or HBEC-3KT epithelial cells, were placed into the p35 dishes. The cocultures were maintained in ALI (Air-Liquid Interface) media consisting of 1 : 1 KSF and DMEM supplemented with 10% FCS and further modified as described in [30]. The incubation time before analysis was three days.

### 2.3. Irradiation

The chronic exposures (1.4 or 14 mGy/h) were implemented in a sterile cell culture incubator (Sanyo, Japan) equipped with a ^137^Cs source at the Centre for Radiation Protection Research, The Wenner-Gren Institute, Stockholm University. The total doses applied were 0.1 Gy (1.4 mGy/h) and 1 Gy (14 mGy/h) as both took 72 hrs. A separate Sanyo incubator was used for nonexposed cells. Three biological replicates were collected from each dose and treatment. The experiments were repeated two or three times as noted in the figure legends.

### 2.4. Immunofluorescence Staining

The cells were plated on membrane inserts on the starting day of the irradiation and cultured for three days until the cumulated dose reached 0.1 or 1 Gy. The cells were fixed with 4% paraformaldehyde for 20 min at room temperature (RT). The blocking was done with 5% FCS (GIBCO) and 0.3% Triton X-100 (Sigma-Aldrich) in PBS at RT for 30 min. The primary antibodies were added to the cells diluted in 1% FCS in 0.3% Triton-X-100-PBS: anti-vimentin (monoclonal mouse anti-human #V6630, Sigma-Aldrich) 1 : 200 for 1 h at RT; anti-E-cadherin 1 : 100 (monoclonal rabbit anti-human #3195, Cell Signaling, Denver, MA, USA) for overnight at 4°C; and anti-fibronectin 1 : 20 (polyclonal sheep anti-human #AF1918, R&D Systems, Minneapolis, MN, USA), again overnight at 4°C. The incubation with secondary Alexa-488-conjugated antibodies (Invitrogen, Carlsbad, CA, USA) was performed for 1 h at room temperature. The cells were mounted with propidium iodide containing antifading media Vectashield (Vector Laboratories, Burlingame, CA, USA). Imaging was performed with a Zeiss AxioImager Z1 fluorescence microscope using AxioVision image analysis software (Carl Zeiss, Göttingen, Germany). The EMT status of the immunofluorescence images was evaluated via blind scoring from two independent researchers following clear scoring criteria and TGF-*β*-treated samples as positive controls. The criteria for EMT-positive were increase in cell size and cell elongation, combined with (1) relocalization of vimentin (BEAS-2B cells) or (2) loss of E-cadherin (HBEC-3KT). The results from the blind scoring were combined and reported. For the cell size measurements, we used the manual cell analysis tool from the ImageJ 1.04 (National Institutes of Health, Bethesda, MD, USA, http://imagej.nih.gov/ij/, 1997-2016) software. Briefly, the cells were marked manually and included in the Region Of Interest (ROI) tool. The measure function from the ROI menu was used to create a table including the area information of each cell. One hundred cells were analyzed from each condition.

### 2.5. Western Blotting

On day 3 after the irradiation onset when the planned dose (0.1 or 1 Gy) has been cumulated, the cells were lysed in buffer containing 8 M Urea, 1 M Thiourea, 30 mM Tris, 4% Chaps buffer 1% protease inhibitor cocktail (Roche, Mannheim, Germany), and 1% phosphatase inhibitor mixture (Protein phosphatase inhibitor cocktail 2 and 3, Sigma-Aldrich). 10 *μ*g of the protein solution was loaded per lane on 4-12% Bis-Tris Amersham precast gels (GE Healthcare Bio-Sciences, Uppsala, Sweden). The separated proteins were transferred onto a PVDF membrane (Bio-Rad Laboratories, Hertfordshire, UK). After incubating with the blocking buffer (5% skimmed milk, 0.5% Tween-20 in PBS or 5% BSA in 0.75% PBS-Tween-20) for 1 h at RT, the membranes were blotted with antibodies against fibronectin (polyclonal sheep anti-human #AF1918, R&D Systems) 1 : 30 000; E-cadherin (monoclonal rabbit anti-human #3195, Cell Signaling) 1 : 5 000; *α*-SMA 1 : 5 000 (monoclonal mouse anti-human #A5228, Sigma-Aldrich); TGF-*β*1 1 : 5000 (polyclonal rabbit anti-human #NB100-91995, Novus Biologicals, Littleton, CO, USA); and GAPDH 1 : 50 000 (monoclonal mouse anti-human #G8795, Sigma-Aldrich) overnight at 4°C. The blots were incubated with secondary HRP-conjugated antibodies (GE Healthcare) for 1 h at room temperature followed by treatment with SuperSignal ECL (Thermo Fisher Scientific, Rockford, IL, USA) and developed on an X-ray sensitive film (GE Healthcare, Buckinghamshire, UK). Protein expression was quantified using the semiquantitative gel analysis function of the ImageJ 1.04 (National Institutes of Health, Bethesda, MD, USA, http://imagej.nih.gov/ij/, 1997-2016) software. The results of each individual protein were normalized to the GAPDH expression.

### 2.6. 8-oxo-dG Analysis

For the 8-oxo-dG analysis, medium was collected simultaneously with cell harvesting and kept frozen at -20°C. The 8-oxo-dG was measured using a modified ELISA assay as described previously [31, 32]. The ELISA kit was provided by Health Biomarkers Sweden AB. Briefly, 1 ml medium was filtered once using a C18 solid phase extraction column (Varian, CA) as described previously [33]. This step is necessary to remove any products other than the 8-oxo-dG that could cross-react with the monoclonal antibody that was used in ELISA. The purified samples were freeze-dried and reconstituted in PBS. 90 *μ*l of the purified sample was mixed with 50 *μ*l of a primary antibody against 8-oxo-dG (Japan Institute for the Control of Aging, Japan) and transferred to a 96-well ELISA plate precoated with 8-oxo-dG. After overnight incubation at 4°C, the plates were washed three times with 250 *μ*l of washing solution. 140 *μ*l of HRP-conjugated secondary antibody (goat anti-mouse IgG-HRP, Scandinavian Diagnostic Services, Sweden) was added to each well and incubated for 2 hours at room temperature. The wells were washed three times with 250 *μ*l of washing solution and finally with PBS (pH 7.4). Then, 140 *μ*l of tetramethylbenzidine liquid substrate (ICN Biomedicals Inc., USA) was added to each well, and the plate was incubated for 15 min at room temperature. The reaction was terminated by adding 70 *μ*l of 2 M H_3_PO_4_ (Merck, Germany). The absorbance was read at 450 nm using an automatic ELISA plate reader (UV-160A, Shimadzu, Kyoto, Japan). Each sample was analyzed in triplicate (variation = 2.5%), and the samples of each experiment were analyzed using the same 96-well ELISA plate. A standard curve for 8-oxo-dG (0.05-10 ng/ml) was established for each plate covering the range of 8-oxo-dG in the samples. The validation of the modified ELISA method was performed by HPLC-EC (*r*^2^: 0.87, *p* < 0.05) as described previously [32]. The comparisons between the ELISA and the HPLC-EC methods showed a linear correlation at the concentration range found in the human blood serum [32]. There was no correlation between the ELISA and the HPLC-EC results when unfiltered samples were used.

### 2.7. Statistical Analysis

Differences between groups were analyzed using paired two-sample Student's *t*-test, part of Excel, MS Office 2003 package, with nonequal assumption of variances or with one-way ANOVA, and Tukey's posttest, part of the statistical package of GraphPad Prism 4 (GraphPad, La Jolla, USA) as noted in the text.

## 3. Results

### 3.1. Protracted *γ*-Radiation with a Dose Rate of 14 mGy/h (1 Gy Total Dose) Induces Changes Connected with EMT in BEAS-2B and HBEC-3KT Bronchial Epithelial Cell Lines

A low-dose rate irradiation facility was used to perform a series of protracted irradiation experiments with two bronchial epithelial cell lines (BEAS-2B and HBEC-3KT). The cells were plated on membrane inserts and exposed to either 1.4 or 14 mGy/h up to 0.1 or 1 Gy cumulated doses, respectively, over a time period of three days as described in Materials and Methods. The cell lines were immunostained for vimentin (BEAS-2B) and E-cadherin (HBEC-3KT). These markers were selected after the two lines were screened for sensitivity to EMT-inducing stimuli and EMT marker expression levels [34]. BEAS-2B cells showed significant changes in vimentin expression and HBEC-3KT in E-cadherin expression, and these two markers were used in [34] and further as the most sensitive.

**Figure 1:**
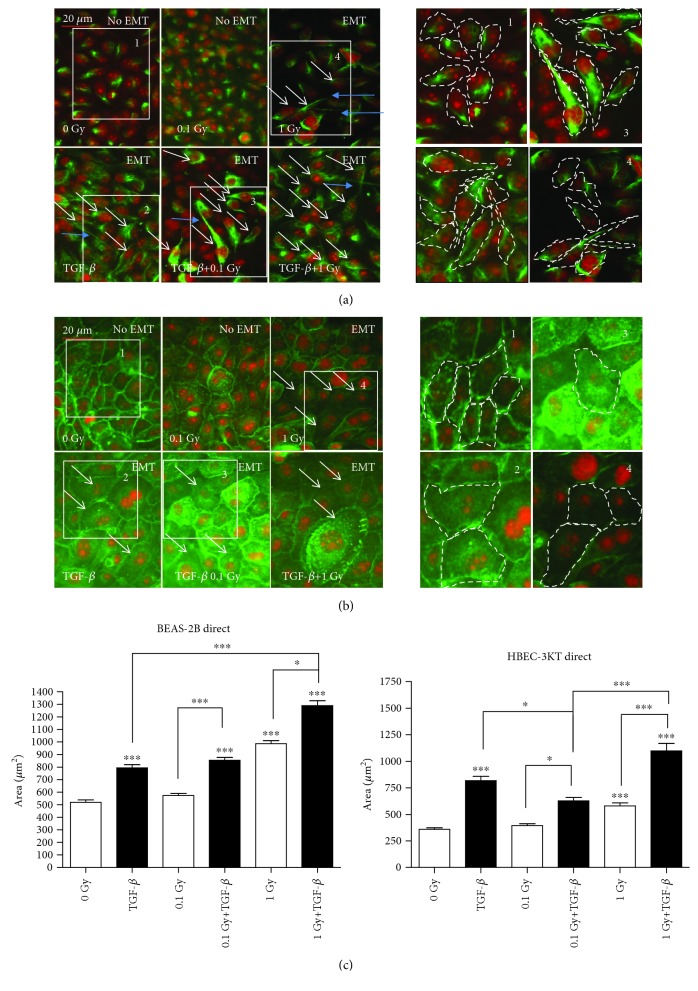
Immunofluorescence staining of EMT marker expression in lung epithelial cell lines: (a) in BEAS-2B with vimentin staining and (b) in HBEC-3KT with E-cadherin staining. The cells were exposed to 0.1 or 1 Gy protracted radiation at a dose rate of 1.4 or 14 mGy/h, upper panels. Lower panels: positive TGF-*β* control and EMT enhancement after combined treatment of the cells with TGF-*β* and 0.1 or 1 Gy of protracted radiation. Vimentin and E-cadherin are stained in green. The nuclei are counterstained with propidium iodide (red). White arrows indicate cells with changes consistent with EMT. Cytoplasmic protrusions are marked with blue arrows. The enlarged same size areas on the right side of (a) for vimentin and (b) for E-cadherin. Numbers 1-4 are visualising the change in cell shape and size: (1) control, (2) TGF-*β*, (3) TGF-*β*+0.1 Gy, and (4) 1 Gy. Scale bars: 20 *μ*m. (c) ImageJ measurements of the cell size in the BEAS-2B and HBEC-3KT cells from the image series (a, b). ^∗^*p* < 0.05 and ^∗∗∗^*p* < 0.001; one-way ANOVA and Tukey's posttest (*n* = 3).

After exposure to protracted irradiation, we observed EMT-related changes (while vimentin levels increase and extend throughout the cytoplasm relative to control, the E-cadherin decreases at the plasma membrane relative to control) in both cell lines after treatment with a dose rate of 14 mGy/h and a total dose of 1 Gy (Figure 1). No EMT and change in the cell size were seen after treatment with 1.4 mGy/h (0.1 Gy total dose). With the higher applied dose (1 Gy), we observed both EMT and a statistically significant increase in the cell size compared to the control. The results show that the mesenchymal marker vimentin was relocalized in BEAS-2B cells undergoing EMT (Figure 1(a)). In the nontreated BEAS-2B cells, vimentin was forming a dense spot in the cytoplasm, while in the cells that had undergone EMT-consistent changes, vimentin was forming a fine intermediate filament network. The BEAS-2B cells changed from an epithelial cuboidal morphology to the elongated form of mesenchymal cells. Interestingly, the cells were exhibiting long cytoplasmic protrusions post irradiation (Figure 1(a), blue arrows). The right panels in Figure 1(a) visualise the changes in cell size and shape during the EMT process in BEAS-2B cells. The inserts have the same size; however, in EMT-positive fields 2, 3, and 4, they contain fewer but larger cells with an elongated shape. We have performed measurements of the elongation of the cells (Suppl. [Supplementary-material supplementary-material-1]). The white arrows show the level of cells which undergone EMT changes in each panel. The areas were selected to represent EMT negative control, positive control (TGF-*β*), irradiated only cells (EMT-positive), and irradiated plus TGF-*β*-treated cells (EMT-positive). When the cell area of the BEAS-2B cells was measured, there was a statistically significant increase in the size post 1 Gy compared to the nontreated controls (Figure 1(c), BEAS-2B graph).

In HBEC-3KT cells, the epithelial marker E-cadherin was decreasing in the cell-to cell-contacts in some, but not all cells. In addition, we observed changes in the cell size in the HBEC-3KT cells as marked in the right side panels containing again the same size insets (Figure 1(b), 1–4). At confluence before the exposure, the cells were small with cobblestone epithelial morphology (Figure 1(b), “No EMT” panels), while after irradiations, they had grown to large (Figure 1(b), “EMT” panels, white arrows) cells. The enlarged areas help to compare the cell size and shape changes between the control (Figure 1(b), 1) and 1 Gy irradiated cells (Figure 1(b), 4). We also performed the measurement of the cell size for the HBEC-3KT cells (Figure 1(c), HBEC-3KT graph). The results were similar as for the BEAS-2B, there are no increase of the size at 0.1 Gy and statistically significant increase at 1 Gy, compared to the control.

In addition to chronic irradiation, we treated the cells with a minimal EMT-inducing concentration of TGF-*β* (0.1-0.2 ng/ml) and the same protracted doses of ionizing radiation at dose rates of 1.4 and 14 mGy/h (total dose 0.1 and 1 Gy, respectively) (Figures 1(a) and 1(b), lower images). In this experimental setup, where we investigated the potentiating effect of radiation on TGF-*β*-induced EMT, a statistically significant additive effect was observed only with 1 Gy radiation for the BEAS-2B cells (Figure 1(c)).

**Figure 2:**
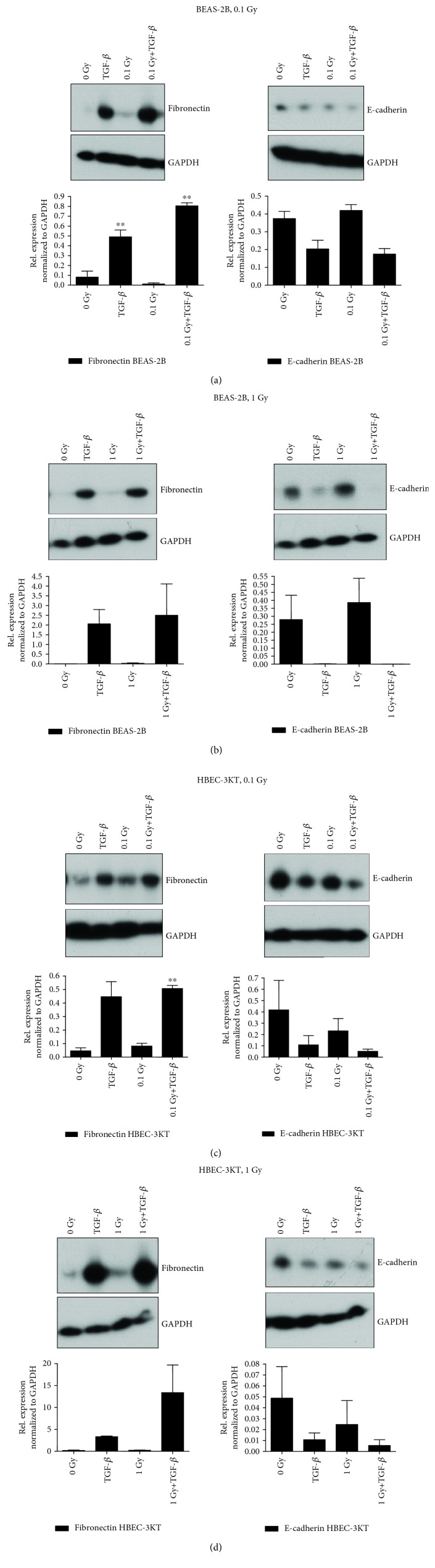
Western blotting analyses of EMT marker expression (a, b) in BEAS-2B and (c, d) HBEC-3KT cells chronically irradiated to a total dose of (a, c) 0.1 (at 1.4 mGy/h) or (b, d) 1 Gy (at 14 mGy/h). Representative western blot analyses of fibronectin expression (a–d, left side blots) and E-cadherin expression (a–d, right side blots). The graphs below are results of quantification of the protein expression as described in Materials and Methods. Error bars—standard deviation, comparison to untreated control. ^∗∗^*p* < 0.01, one-way ANOVA and Tukey's posttest (*n* = 3).

While the effect of irradiation on the expression and relocalization of the EMT markers was clear when detected by immunofluorescence, western blotting and semiquantitative evaluation show significant changes to the control only at 0.1 Gy in BEAS-2B cells and 0.1 Gy+TGF-*β* treatments for the fibronectin marker (Figure 2) for both cell lines. Vimentin is a good marker for immunofluorescence studies but not expected to be quantitative for western blots because the concentration of the vimentin protein is not changed, rather distribution within the cytoplasm is more diffused during EMT. That is why we have compared only E-cadherin and fibronectin in the western blots.

Although the analysis failed to show a statistical significance in the majority of the western blot analyses (except BEAS-2B 0.1 Gy and 0.1 Gy+TGF-*β* and HBEC-3KT 0.1 Gy+TGF-*β* for fibronectin), a trend characteristic for EMT (increase of fibronectin and decrease of E-cadherin levels) and also enhancement of the TGF-*β*-induced EMT in combination with the IR (Figure 2) were observed in the HBEC-3KT cells. The other cell line BEAS-2B responded with EMT characteristic changes in the fibronectin and E-cadherin only to TGF-*β* ± irradiation.

### 3.2. Effect of the Microenvironment on EMT Characteristic Changes in Fibroblast-Epithelial Cell Cocultures

**Figure 3:**
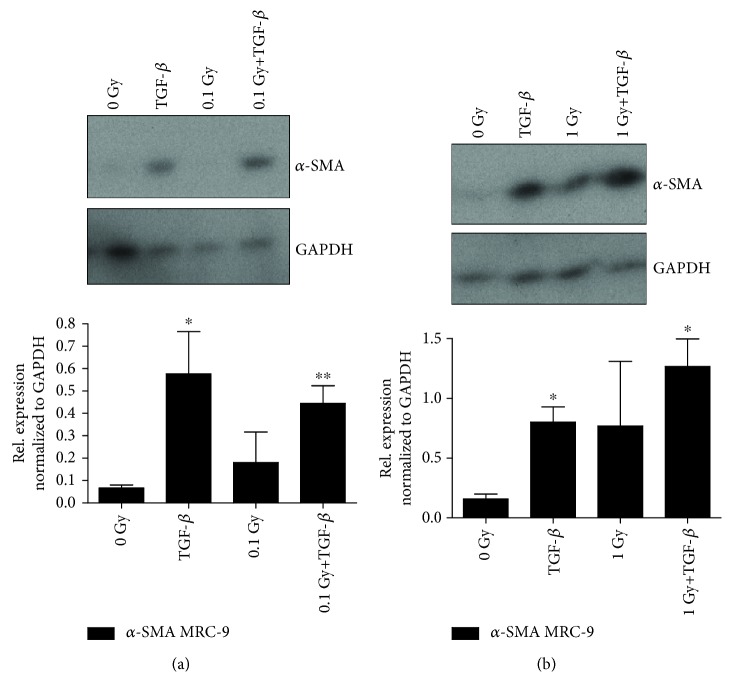
Myofibroblastic marker *α*-SMA expression in MRC-9 fibroblasts post chronic low LET irradiation at dose rates of (a) 1.4 mGy/h to 0.1 Gy or (b) 14 mGy/h to 1 Gy with or without TGF-*β*. The graphs below are the results of the semiquantification of the protein expression as described in Materials and Methods. Error bars—standard deviation. ^∗^*p* < 0.05 and ^∗∗^*p* < 0.01; one-way ANOVA and Tukey's posttest (*n* = 3).

The microenvironment is involved in the TGF-*β*-induced EMT process via secretion of signaling molecules from the stromal cells [35, 36]. We observed that TGF-*β* induces higher expression of *α*-SMA, an actin isoform involved in the formation of stress fibers during myofibroblastic differentiation in MRC-9 cells (Figure 3) [7, 11]. There is also a trend in increased *α*-SMA with 1 Gy ionizing radiation in the MRC-9 cells.

As shown in Figure 3(a), a dose of 0.1 Gy delivered at 1.4 mGy/h was able to increase slightly the *α*-SMA expression. The 1 Gy exposures induced a higher level of the stress fiber protein in comparison to the 0.1 Gy (Figure 3). The combination of TGF-*β* treatment and 1 Gy radiation exposure had some cumulative effects on the *α*-SMA expression compared to the TGF-*β* only. However, we have to point that we could not show a statistically significant increase of the radiation and TGF-*β* effects in comparison to the TGF-*β* treatments alone (Figure 3).

**Figure 4:**
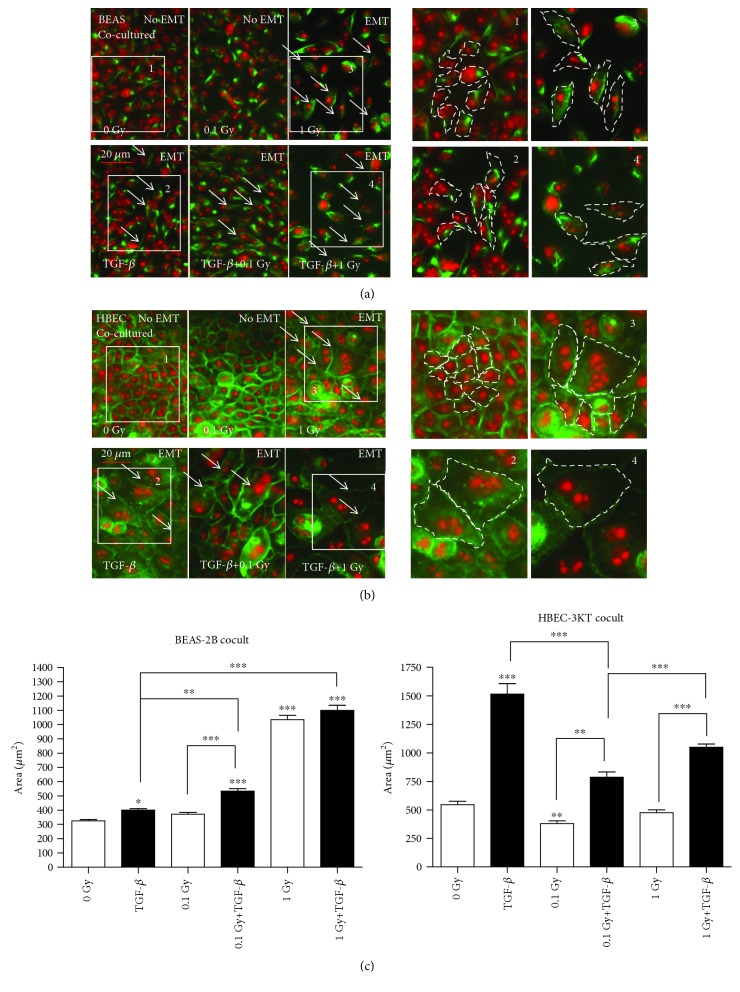
Immunofluorescence staining of EMT marker expression in lung epithelial cell lines cocultured with MRC-9 fibroblasts: (a) vimentin in BEAS-2B and (b) E-cadherin in HBEC-3KT. The cocultures were exposed to 0.1 or 1 Gy of protracted radiation at the dose rates of 1.4 or 14 mGy/h, upper panels. Lower panels: positive control TGF-*β* treatment and the EMT enhancement after the combined treatment of the TGF-*β*+0.1 cells or 1 Gy of protracted radiation. Vimentin and E-cadherin are stained in green. The nuclei are counterstained with propidium iodide (red). White arrows indicate the cells with consistent changes. Scale bars: 20 *μ*m. The enlarged same size areas on the right side of (a) for vimentin and (b) for E-cadherin with numbers 1-4 are visualising the change in cell shape and size: (1) control, (2) TGF-*β*, (3) 1 Gy, and (4) TGF-*β*+1 Gy. (c) ImageJ measurements of the cell size in the BEAS-2B and HBEC-3KT cells from the image series (a, b). ^∗^*p* < 0.05, ^∗∗^*p* < 0.01, and ^∗∗∗^*p* < 0.001; one-way ANOVA and Tukey's posttest (*n* = 3).

In coculturing experiments the epithelial (BEAS-2B or HBEC-3KT) cells were plated in membrane inserts while the stromal cells were plated in 35 mm Petri dishes. The cocultures were either sham irradiated or exposed to the same cumulative doses of 0.1 and 1 Gy at 1.4 or 14 mGy/h, respectively. To test if the coculture ALI medium that contains 5% fetal calf serum (potentially containing minimal quantities of TGF-*β*) induces the EMT changes in the epithelial cells, we had a parallel negative control where the cells were incubated in the ALI medium without a coculture with the MRC-9 fibroblast. As shown in Suppl. [Supplementary-material supplementary-material-1], the ALI medium was not able to induce the EMT. At the same time, the coculture and the same exposure time of 14 mGy/h irradiation had a higher EMT-inducing effect than radiation only (Figures 1 and 4).

In the BEAS-2B cells, enhanced EMT characteristic changes were detected when the cells were cocultured with fibroblasts and irradiated with a dose rate of 14 mGy/h up to a cumulative dose of 1 Gy, in comparison to those irradiated with a dose rate 1.4 mGy/h to a dose of 0.1 Gy over the same time period (Figure 4(a)). With the HBEC-3KT cells, coculturing with fibroblasts enhanced radiation and the TGF-*β*-induced EMT-specific changes with a dose of 1 Gy (Figure 4(b)). Interestingly, the features of the cocultured cells undergoing EMT-like transformation, for both BEAS-2B and HBEC-3KT, are different than those of the monocultures (Figure 4 and Suppl. [Supplementary-material supplementary-material-1]). As could be seen in Figure 4 and the insets (Figures 4(a) and 4(b), 3 and 4), the irradiated and TGF-*β*-treated cells are both larger than control and compared to the TGF-*β* only (Figure 4(c)), and for the HBEC-3KT (Figure 4(b), 3 and 4), they are containing more than one nucleus (Figure 4(b)).

### 3.3. 8-oxo-dG Production from MRC-9, BEAS-2B, and HBEC-3KT Cells

**Figure 5:**
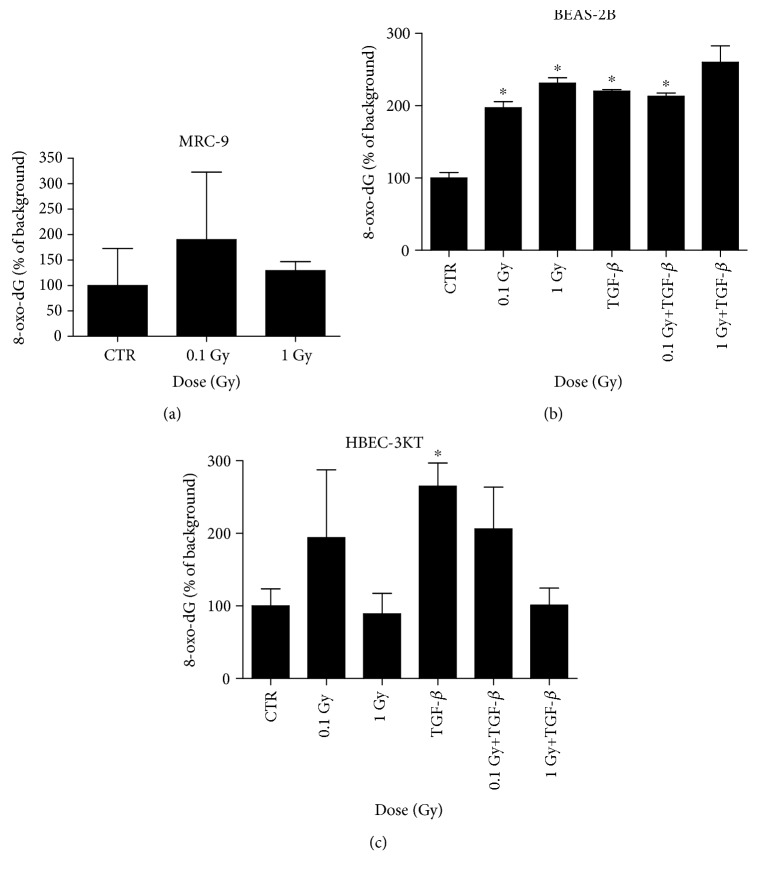
8-oxo-dG secretion in chronically irradiated MRC-9 fibroblast cells (a), (b) BEAS-2B or (c) HBEC-3KT cells. The cells have been exposed to a total of 0.1 (at 1.4 mGy/h) or 1 Gy (14 mGy/h). Error bars—standard deviation. Statistical analysis—Student's *t*-test (*n* = 2) (a). In addition, the same cells were treated with the TGF-*β* in combination to radiation ((b) 0.1 or (c) 0.2 ng/ml) as described in Materials and Methods. Error bars—standard deviation. ^∗^*p* < 0.05; one-way ANOVA and Tukey's posttest (*n* = 2).

Radiation-induced oxidative stress is one of the main cell-damaging conditions induced by low LET radiation such as X- or *γ*-rays. The reactive oxygen species generated during the exposure to radiation in aqueous solutions are able to damage biological molecules, including DNA and cytoplasmic DNA precursors (dNTP). One of the products from the oxidation of the deoxyribonucleotides, 8-oxo-dG, has proved to accumulate after irradiation. Radiation-induced extracellular 8-oxo-dG has been reported even after very low dose and low-dose rate exposures [18, 31]. In our study, chronic irradiation causes the increase of 8-oxo-dG from the MRC-9 fibroblasts in comparison with the background levels (Figure 5(a)). However, the induction was very small and not statistically significant. On the other hand, radiation-induced oxidative stress levels were increased in the chronically irradiated epithelial cell lines as in one of them, BEAS-2B we observed a statistically significant dose-dependent increase (Figure 5(b)).

Also, the combination of TGF-*β* treatment and irradiation had a slight cumulative effect on the 1 Gy delivered at 14 mGy/h. The second cell line, HBEC-3KT, although showing a statistically significant induction of 8-oxo-dG in the TGF-*β* treated cells, failed to respond to radiation or to a combination of radiation and TGF-*β* in the same statistically significant manner (Figure 5(c)). This might be explained with the differences in the genetic background and antioxidant properties of the two cell lines [29].

## 4. Discussion

Based on the data for synergy between TGF-*β* signaling and radiation exposure in a mouse mammary gland model [19, 37], we introduce a new mechanistic model for the lung in which protracted exposure to low-dose radiation activates the stromal TGF-*β* pathway and leads to the radiation-induced EMT of the lung epithelial cells. We have previously reported that low doses of gamma radiation induce oxidative stress in a nonlinear manner [31, 32] with a peak already at doses of about 5 mGy. In our earlier studies, cells exposed to low-dose chronic irradiation have constantly elevated oxidative stress [16]. We have also shown that the main target for radiation-induced oxidative stress is the intracellular nucleotide pool, dNTP, where different types of modified dNTP can be produced, e.g., 8-oxo-dGTP. 8-oxo-dGTP release from cells such as 8-oxo-dG can be detected as a measurement of oxidative stress [31, 32]. In this study, the results suggest that the low-dose rate radiation might induce oxidative stress and that this possibly activates TGF-*β* and leads to EMT-connected cell transformation, even with low cumulative dose exposure (0.1 Gy delivered at 1.4 mGy/h). We detected an increased level of the oxidative markers (8-oxo-dG) in the culture media of the irradiated and double treated (radiation and TGF-*β*) lung epithelial cells. Oxidative stress has been previously described to play a crucial role in the TGF-*β* activation [7, 31, 38]. We hypothesize that the elevated oxidative stress and the TGF-*β* signaling in lung epithelium and microenvironment are co-operating and acting synergistically leading to the observed enhanced EMT-consistent changes particularly for a dose of 1 Gy in the BEAS-2B cell line. In further studies, there should also be considered other important and more sensitive indicators of the TGF-*β* signaling activation such as p-smad 2/3 or the Pai-1 luciferase assay [10–12].

In our study, the additional treatment of the cells with a low concentration of TGF-*β* had a potentiating effect on the protracted low-dose radiation-induced EMT only when the cumulative dose reached 1 Gy for the BEAS-2B cell line. Earlier publications by Ehrhart et al. [39] showed that TGF-*β* is activated after low doses of low LET radiation in the *in vivo* mouse mammary gland model. Although they found a dose-dependent relationship for the activation of the factors, the observed effects were the strongest at the lowest dose applied (0.1 Gy). We observed a similar trend in our experiments for oxidative stress induction in the epithelial cell line (BEAS-2B) at 0.1 Gy. Earlier, we detected an increase in the TGF-*β* levels in the MRC-9 fibroblasts after acute exposure to doses of 0.1-2 Gy [34]. Other scientists have reported a significant role of TGF-*β* in the activation of oxidative stress response, especially in myofibroblastic cells during their invasive transformation [40].

The activation of TGF-*β* signaling in the MRC-9 cells post radiation is plausible as the molecule is one of the main fibroblast activators responsible for their transition into myofibroblasts or CAFs [36, 41]. As TGF-*β* is also an important factor produced by stromal cells post irradiation and involved in cancer progression, it has been widely considered a potential therapeutic target for reducing metastases and formation of secondary cancers after radiotherapy. Blocking its expression or application of functional inhibitors could be a potential therapy for secondary cancer dissemination [42, 43].

We investigated the effects of low-dose protracted radiation in two immortalized bronchial epithelial cell lines, which are commonly used as a model of lung epithelium. An interesting finding after the protracted low-dose rate irradiations was the detection of various EMT features in the epithelial cells that differed after irradiation compared to TGF-*β* induction. For example, the analysis of the BEAS-2B cells showed that the radiation-induced EMT was characterized by an elongated shape with single cilium-like cytoplasmic protrusions (Figure 1(a); 1 Gy, blue arrows), while the typical TGF-*β*-induced EMT phenotype was more spindle-shaped with a well-defined vimentin network (Figure 1(a); TGF-*β*, white arrows). The vimentin behavior could be connected with its functionality—the dense perinuclearly concentrated vimentin in the nontreated cells is nonactive, while the well-spread intermediate filament network has a crucial role for EMT and motility in many mesenchymal type cells [44]. The additive effect of radiation and TGF-*β* was observed only after the higher cumulated dose of 1 Gy for both cell lines. The role of the long protrusions in the irradiated cells is not clear. It is possible that the cells are being transformed into fibroblasts, which described a spindle shape and are expressing a high level of the mesenchymal marker fibronectin. Accumulation of EMT-generated fibroblasts could accelerate the radiation-induced lung fibrosis in agreement with Guarino et al. [25] and Lee and Nelson [13]. This should be studied further in more detail in a different setup that involves *in vivo* experiments.

In the coculturing experiments (Figure 4), we show an increase between TGF-*β* vs. 0.1 Gy+TGF-*β* and TGF-*β* vs. 1 Gy+TGF-*β* EMT-related changes only in BEAS-2B cells. The 8-oxo-dG is increasing in response to chronic irradiation or to TGF-*β* again only in the BEAS-2B cells (Figure 5(b)). However, the two effects do not appear to be additive except to small extent for the 1 Gy+TGF-*β* in the BEAS-2B cells.

In our prestudies with acute radiation exposures, some of the classical features of the EMT were not affected, although some of the markers or phenotypic features were changed. For example, the ZO-1 and *β*-catenin expression were not affected (data not shown), while the vimentin and E-cadherin were, respectively, up- or downregulated [34]. In the earlier study, we could not prove any clear EMT induction with low or moderate doses of *α*-particles and *γ*-rays. Also, no significant additive effect with radiation and TGF-*β* was observed. However, the results from the current study suggest that (1) radiation may induce partial EMT features; (2) the radiation and particularly the protracted radiation-induced EMT potentially have their own distinguishable features; (3) the combination of radiation and TGF-*β* has an additive effect on the EMT, but this particular feature very likely depends on the antioxidative properties of the cell line; and finally (4) stromal/epithelial cell cocultures enhance the induction of EMT in one of the two cell lines studied (BEAS-2B) suggesting that chronic *in vivo* exposures can influence precarcinogenic changes in the normal epithelium via signals from the microenvironment.

In the two epithelial cell lines that have been used, there is a clear difference in the oxidative stress product levels post irradiation (Figures 5(b) and 5(c)). The BEAS-2A cells are more prone to accumulation of 8-oxo-dG than the HBEC-3KT cells. This can be connected to the viral oncoprotein (SV40 large T antigen) immortalization of the BEAS-2B cells [45]. While the immortalized HBEC-3KT cells are not carcinogenic, the BEAS-2B cells are closer in their genetic properties to tumor cells and can undergo malignant transformations after repetitive passaging [29]. The BEAS-2B cells are reported also to have lower antioxidant activities, e.g., for MnSOD [45]. This is compromising their defense against free radicals, leads to elevated extracellular 8-oxo-dG, and explains the dose response relation for radiation-induced extracellular 8-oxo-dG presented in Figure 5(b). For HBEC-3KT, no clear dose response relation for radiation-induced extracellular 8-oxo-dG was observed (Figure 5(c)). The response of HBEC-3KT cells at the levels of radiation-induced 8-oxo-dG in the medium suggests that HBEC-3KT cells might have a problem to release 8-oxo-dGTP from the cytoplasm to the medium as similar pattern of results were previously observed in primary fibroblast knockdown in the hMTH1 (Figure 5(c), [18]).

To our knowledge, there are no other studies describing how a chronic exposure to low doses and dose rates ionizing radiation affects both the microenvironment and the epithelial cells. We focused on the EMT process as one of the potential precarcinogenic stages undergone by the epithelial cells during malignant transformation. What we found was a partial EMT, concerning only some of the EMT features in the epithelial cells, when they were exposed to radiation without the stromal component. Further, some enhanced EMT-consistent changes were monitored in the TGF-*β*-treated and cocultured irradiated BEAS-2B epithelial cells. These observations suggest how the cellular transformation after low-dose radiation exposures depends on the cell-to-cell interactions.

## Data Availability

The data used to support the findings of this study are available from the corresponding author upon request.

## Supplementary Materials

Supplementary Materials Supplementary Figure 1: elongation in lung epithelial cell lines BEAS-2B (A) and HBEC-3KT (B) post chronic irradiation with or without MRC-9 fibroblasts. Elongation factor (cell length by cell diameter) was calculated manually by ImageJ in 50 cells from each sample. Supplementary Figure 2: immunofluorescence staining of vimentin expression in lung epithelial cell line BEAS-2B cocultured with MRC-9 fibroblasts after protracted irradiation at 1.4 and 14 mGy/h to 0.1 or 1 Gy. Negative control without the fibroblast cells is shown on panel 0 Gy/-MRC-9. Supplementary Figure 3: comparison between the EMT markers' expression in monocultures of bronchial epithelial cells (A) BEAS-2B and vimentin and (B) HBEC-3K and E-cadherin upper panels; (A and B, lower panels) and cocultures of the same cells and MRC-9 fibroblasts, after a protracted irradiation at 1.4 and 14 mGy/h to 0.1 or 1 Gy. Vimentin and E-cadherin are stained in green. The nuclei are counterstained with propidium iodide (red). White arrows indicate the EMT undergone cells. Scale bars: 20 *μ*m.
