# Supplementary material for: Presence of Stromal Cells Enhances Epithelial-to-Mesenchymal Transition (EMT) Induction in Lung Bronchial Epithelium after Protracted Exposure to Oxidative Stress of Gamma Radiation
Source: Oxid Med Cell Longev. 2019 Sep 8;2019:4120379. doi: 10.1155/2019/4120379 (PMC6754954; doi:10.1155/2019/4120379)

Supplementary Figure 1. Elongation in lung epithelial cell lines BEAS-2B (A) and HBEC-3KT (B) post chronic irradiation with or without MRC-9 fibroblasts. Elongation factor (cell length by cell diameter) was calculated manually by Image J in 50 cells from each sample.

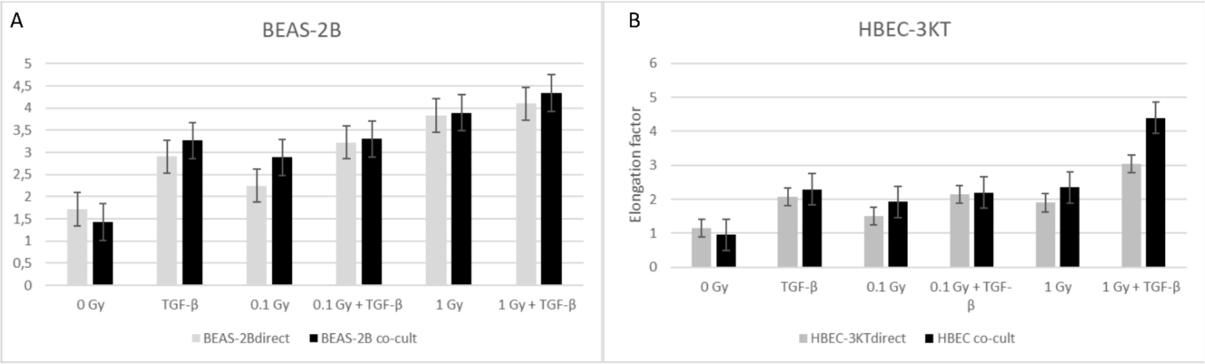

Supplementary Figure 2. Immunofluorescence staining of vimentin expression in lung epithelial cell line BEAS-2B co-cultured with MRC-9 fibroblasts after protracted irradiation at 1.4 and 14 mGy/h to 0.1 or 1 Gy. Negative control without the fibroblast cells is shown on panel 0 Gy/ - MRC-9.

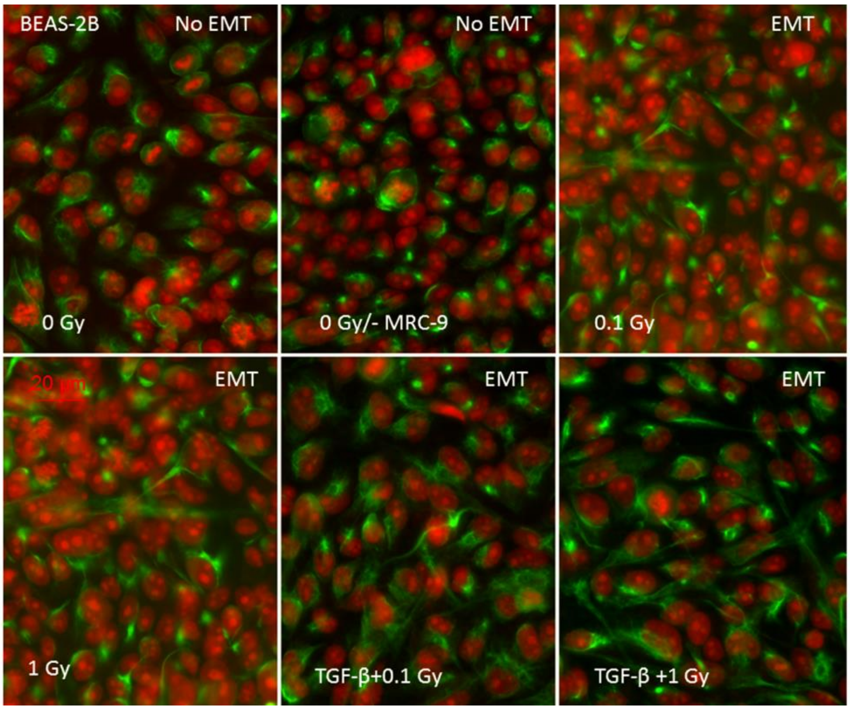

Supplementary Figure 3. Comparison between the EMT markers' expression in monocultures of bronchial epithelial cells (A) BEAS-2B, vimentin; and (B) HBEC-3K, E-cadherin upper panels; (A and B, lower panels) and co-cultures of the same cells and MRC-9 fibroblasts, after a protracted irradiation at 1.4 and 14 mGy/h to 0.1 or 1 Gy. Vimentin and E-cadherin are stained in green. The nuclei are counterstained with propidium iodide (red). White arrows indicate the EMT-undergone cells. Scale bars - 20 µm.

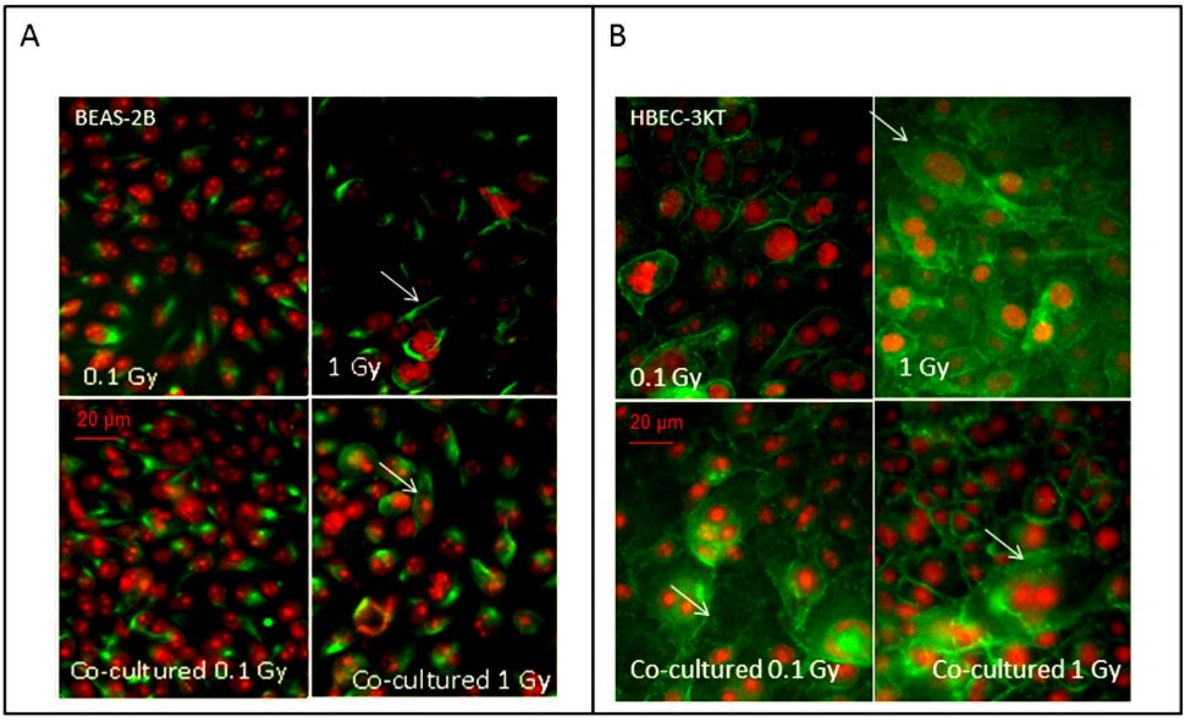

Supplement: Supplementary Materials — Supplementary Figure 1: elongation in lung epithelial cell lines BEAS-2B (A) and HBEC-3KT (B) post chronic irradiation with or without MRC-9 fibroblasts. Elongation factor (cell length by cell diameter) was calculated manually by ImageJ in 50 cells from each sample. Supplementary Figure 2: immunofluorescence staining of vimentin expression in lung epithelial cell line BEAS-2B cocultured with MRC-9 fibroblasts after protracted irradiation at 1.4 and 14 mGy/h to 0.1 or 1 Gy. Negative control without the fibroblast cells is shown on panel 0 Gy/-MRC-9. Supplementary Figure 3: comparison between the EMT markers' expression in monocultures of bronchial epithelial cells (A) BEAS-2B and vimentin and (B) HBEC-3K and E-cadherin upper panels; (A and B, lower panels) and cocultures of the same cells and MRC-9 fibroblasts, after a protracted irradiation at 1.4 and 14 mGy/h to 0.1 or 1 Gy. Vimentin and E-cadherin are stained in green. The nuclei are counterstained with propidium iodide (red). White arrows indicate the EMT undergone cells. Scale bars: 20 μm. [file 4120379.f1.pdf]
